# 4,6-Dibromo­isophthalic acid monohydrate

**DOI:** 10.1107/S1600536812033892

**Published:** 2012-08-04

**Authors:** Bao-fen Ye

**Affiliations:** a Department of Analytical Chemistry, China Pharmaceutical University, Nanjing 210096, People’s Republic of China

## Abstract

In the crystal structure of the title hydrate, C_8_H_4_Br_2_O_4_·H_2_O, O—H⋯O hydrogen bonds link the mol­ecules into a two-dimensional network parallel to (10-2). The acid groups of the main mol­ecule and the water mol­ecule are all involved in the supra­molecular structure. The dihedral angles between the benzene ring and the acid groups are 37.8 (4) and 36.4 (5)°, while the dihedral angle between the acid groups is 10.9 (4)°.

## Related literature

For the synthesis of the title compound, see: Singh & Bedi (1957 ▶). For a related structure, see: Song *et al.* (2008 ▶).
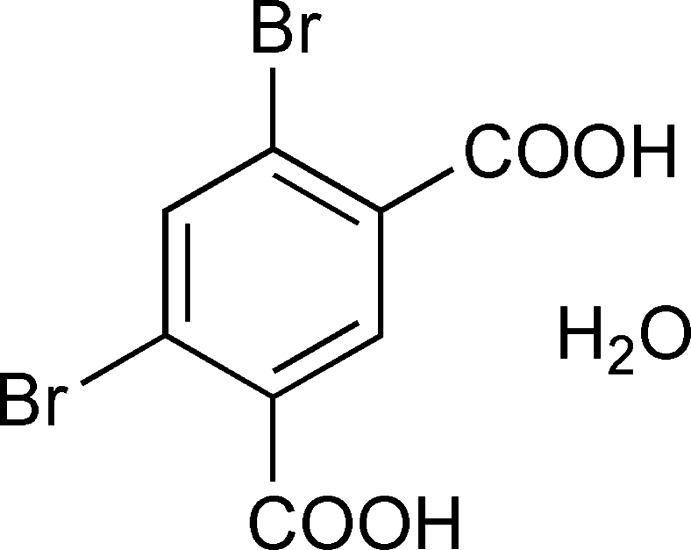


## Experimental

### 

#### Crystal data

C_8_H_4_Br_2_O_4_·H_2_O
*M* *_r_* = 341.95
Monoclinic, 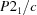

*a* = 3.8740 (8) Å
*b* = 17.366 (4) Å
*c* = 15.710 (3) Å
β = 90.91 (3)°
*V* = 1056.8 (4) Å^3^
*Z* = 4
Mo *K*α radiation
μ = 7.67 mm^−1^
*T* = 293 K
0.30 × 0.05 × 0.05 mm

#### Data collection

Enraf–Nonius CAD-4 diffractometer
Absorption correction: ψ scan (North *et al.*, 1968 ▶) *T*_min_ = 0.207, *T*_max_ = 0.700
2206 measured reflections
1910 independent reflections
1109 reflections with *I* > 2σ(*I*)
*R*_int_ = 0.051
3 standard reflections every 200 reflections intensity decay: none

#### Refinement

*R*[*F* ^2^ > 2σ(*F* ^2^)] = 0.054
*wR*(*F* ^2^) = 0.089
*S* = 0.99
1910 reflections
136 parameters
H-atom parameters constrained
Δρ_max_ = 0.63 e Å^−3^
Δρ_min_ = −0.50 e Å^−3^

### 

Data collection: *CAD-4 Software* (Enraf–Nonius, 1985 ▶); cell refinement: *CAD-4 Software*; data reduction: *XCAD4* (Harms & Wocadlo, 1995 ▶); program(s) used to solve structure: *SHELXS97* (Sheldrick, 2008 ▶); program(s) used to refine structure: *SHELXL97* (Sheldrick, 2008 ▶); molecular graphics: *SHELXTL* (Sheldrick, 2008 ▶); software used to prepare material for publication: *SHELXTL*.

## Supplementary Material

Crystal structure: contains datablock(s) I, global. DOI: 10.1107/S1600536812033892/bh2450sup1.cif

Structure factors: contains datablock(s) I. DOI: 10.1107/S1600536812033892/bh2450Isup2.hkl

Supplementary material file. DOI: 10.1107/S1600536812033892/bh2450Isup3.cml

Additional supplementary materials:  crystallographic information; 3D view; checkCIF report

## Figures and Tables

**Table 1:** Hydrogen-bond geometry (Å, °)

*D*—H⋯*A*	*D*—H	H⋯*A*	*D*⋯*A*	*D*—H⋯*A*
O2—H2*A*⋯O*W*	0.82	1.74	2.554 (8)	176
O4—H4*B*⋯O1^i^	0.82	1.86	2.665 (8)	168
O*W*—H*WB*⋯O3^ii^	0.85	2.08	2.893 (9)	159
O*W*—H*WA*⋯O3^iii^	0.85	2.17	2.903 (9)	144

Symmetry codes: (i) 
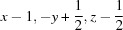
; (ii) 
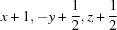
; (iii) 
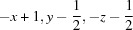
.

**Fig. 1.**
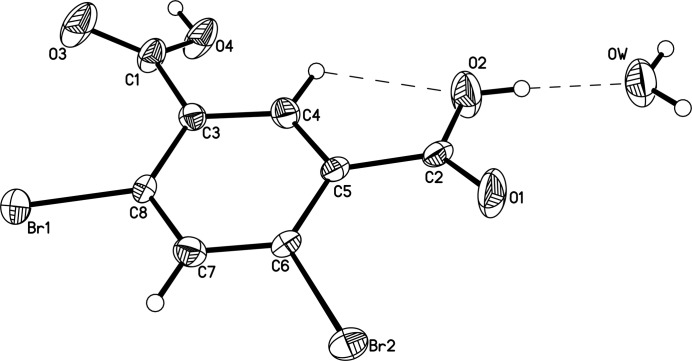
The molecular structure of the title compound, with displacement ellipsoids at the 30% probability level. Dashed lines are non-bonding contacts.

**Fig. 2.**
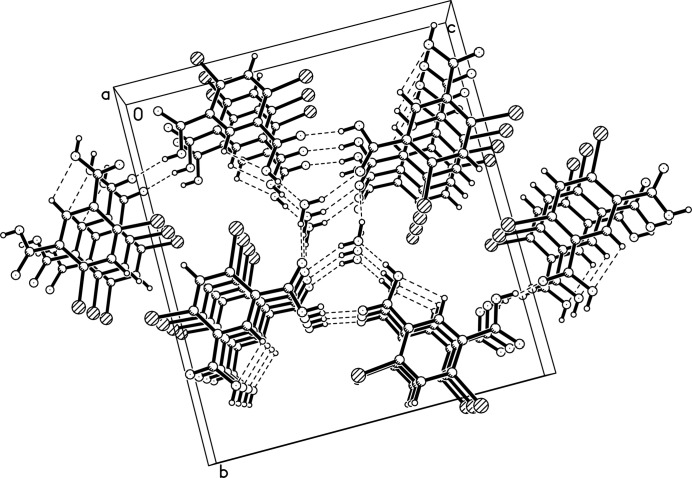
A packing diagram for the title compound, with O—H···O intermolecular hydrogen bonds shown as dashed lines.

## supplementary crystallographic information

### Comment

4,6-Dibromoisophthalic acid (DBPA) is an important organic intermediate for organic synthesis, which can be used in many fields such as organic light-emitting materials. We report herein the crystal structure of the hydrate of DBPA (Fig. 1). Bond lengths and angles are within normal ranges (Song *et al.*, 2008). The asymmetric unit contains one 4,6-dibromoisophthalic acid molecule and one water molecule, in general positions. In the crystal, O—H···O hydrogen bonds link the molecules to form a bidimensional framework (Fig. 2), where all OH groups of the DBPA and the water molecules are involved, as well as carbonyl groups. The water molecule serves as donor and acceptor for hydrogen bonding.

### Experimental

DBPA was prepared according to the literature method (Singh & Bedi, 1957). Single crystals of the title hydrate suitable for X-ray analysis were obtained by dissolving DBPA (2.0 g) in water (80 ml) and evaporating the solution slowly at room temperature for about 15 days.

### Refinement

All H atoms may be found in a difference map, but their positions were fixed in the final refinement with idealized bond lengths of 0.82 (OH of acid), 0.85 (water molecule) or 0.93 Å (aromatic CH). Isotropic displacement parameters for H atoms were calculated as *U*_iso_(H) = *xU*_eq_(parent atom), where *x* = 1.5 for acid OH groups, and *x* = 1.2 for other H atoms.

### Crystal data

**Table:**

C_8_H_4_Br_2_O_4_·H_2_O	*F*(000) = 656
*M**_r_* = 341.95	*D*_x_ = 2.149 Mg m^−^^3^
Monoclinic, *P*2_1_/*c*	Melting point = 441–443 K
Hall symbol: -P 2ybc	Mo *K*α radiation, λ = 0.71073 Å
*a* = 3.8740 (8) Å	Cell parameters from 25 reflections
*b* = 17.366 (4) Å	θ = 10–14°
*c* = 15.710 (3) Å	µ = 7.67 mm^−^^1^
β = 90.91 (3)°	*T* = 293 K
*V* = 1056.8 (4) Å^3^	Rodlike, colourless
*Z* = 4	0.30 × 0.05 × 0.05 mm

### Data collection

**Table:**

Enraf–Nonius CAD-4 diffractometer	1109 reflections with *I* > 2σ(*I*)
Radiation source: fine-focus sealed tube	*R*_int_ = 0.051
Graphite monochromator	θ_max_ = 25.2°, θ_min_ = 1.8°
ω/2θ scans	*h* = −4→4
Absorption correction: ψ scan (North *et al.*, 1968)	*k* = 0→20
*T*_min_ = 0.207, *T*_max_ = 0.700	*l* = 0→18
2206 measured reflections	3 standard reflections every 200 reflections
1910 independent reflections	intensity decay: none

### Refinement

**Table:**

Refinement on *F*^2^	Primary atom site location: structure-invariant direct methods
Least-squares matrix: full	Secondary atom site location: difference Fourier map
*R*[*F*^2^ > 2σ(*F*^2^)] = 0.054	Hydrogen site location: inferred from neighbouring sites
*wR*(*F*^2^) = 0.089	H-atom parameters constrained
*S* = 0.99	*w* = 1/[σ^2^(*F*_o_^2^) + (0.02*P*)^2^] where *P* = (*F*_o_^2^ + 2*F*_c_^2^)/3
1910 reflections	(Δ/σ)_max_ < 0.001
136 parameters	Δρ_max_ = 0.63 e Å^−^^3^
0 restraints	Δρ_min_ = −0.50 e Å^−^^3^
0 constraints	

### Fractional atomic coordinates and isotropic or equivalent isotropic displacement parameters (Å^2^)

**Table:**

	*x*	*y*	*z*	*U*_iso_*/*U*_eq_	
OW	0.6960 (16)	0.0557 (3)	−0.0592 (4)	0.079 (2)	
HWB	0.8013	0.0536	−0.0114	0.094*	
HWA	0.6946	0.0116	−0.0828	0.094*	
Br1	−0.1479 (2)	0.52659 (5)	−0.24930 (5)	0.0486 (3)	
O1	0.6060 (18)	0.2282 (4)	−0.0212 (4)	0.073 (2)	
C1	−0.041 (2)	0.3595 (5)	−0.3421 (5)	0.041 (2)	
Br2	0.3840 (2)	0.38158 (6)	0.03673 (5)	0.0473 (3)	
C2	0.4568 (19)	0.2307 (5)	−0.0891 (5)	0.0314 (19)	
O2	0.4320 (16)	0.1710 (3)	−0.1373 (4)	0.071 (2)	
H2A	0.5248	0.1342	−0.1136	0.107*	
C3	0.0622 (17)	0.3658 (4)	−0.2523 (5)	0.0297 (19)	
O3	−0.0053 (17)	0.4099 (4)	−0.3936 (4)	0.070 (2)	
C4	0.1931 (18)	0.3018 (5)	−0.2089 (5)	0.034 (2)	
H4A	0.2057	0.2556	−0.2385	0.041*	
O4	−0.1724 (15)	0.2937 (4)	−0.3616 (3)	0.0598 (18)	
H4B	−0.2295	0.2935	−0.4121	0.090*	
C5	0.3038 (18)	0.3022 (5)	−0.1261 (4)	0.0294 (19)	
C6	0.2627 (17)	0.3713 (5)	−0.0796 (4)	0.0315 (19)	
C7	0.1313 (18)	0.4354 (5)	−0.1184 (5)	0.036 (2)	
H7A	0.1089	0.4807	−0.0873	0.043*	
C8	0.0319 (18)	0.4341 (4)	−0.2022 (4)	0.0293 (18)	

### Atomic displacement parameters (Å^2^)

**Table:**

	*U*^11^	*U*^22^	*U*^33^	*U*^12^	*U*^13^	*U*^23^
OW	0.131 (6)	0.051 (4)	0.053 (4)	0.036 (4)	−0.027 (4)	−0.007 (4)
Br1	0.0605 (6)	0.0433 (5)	0.0422 (5)	0.0132 (5)	0.0028 (4)	0.0097 (5)
O1	0.131 (6)	0.049 (4)	0.039 (4)	0.029 (4)	−0.031 (4)	0.003 (3)
C1	0.057 (6)	0.040 (6)	0.027 (5)	−0.004 (5)	−0.007 (4)	0.006 (5)
Br2	0.0647 (6)	0.0517 (6)	0.0254 (4)	0.0066 (5)	−0.0047 (4)	−0.0072 (5)
C2	0.036 (5)	0.039 (5)	0.019 (4)	0.000 (4)	0.003 (4)	0.001 (4)
O2	0.126 (6)	0.038 (4)	0.050 (4)	0.023 (4)	−0.025 (4)	−0.006 (4)
C3	0.029 (5)	0.032 (5)	0.028 (4)	0.004 (4)	0.000 (3)	0.000 (4)
O3	0.125 (6)	0.061 (5)	0.024 (3)	−0.023 (4)	−0.011 (3)	0.006 (4)
C4	0.039 (5)	0.025 (4)	0.038 (5)	−0.002 (4)	0.002 (4)	−0.003 (4)
O4	0.097 (5)	0.056 (5)	0.026 (3)	−0.024 (4)	−0.014 (3)	0.005 (3)
C5	0.035 (5)	0.031 (5)	0.022 (4)	0.000 (4)	0.007 (4)	0.002 (4)
C6	0.038 (5)	0.037 (5)	0.020 (4)	0.001 (4)	0.005 (3)	0.004 (4)
C7	0.037 (5)	0.036 (5)	0.034 (5)	0.002 (4)	0.001 (4)	−0.005 (4)
C8	0.038 (5)	0.026 (4)	0.024 (4)	0.001 (4)	0.003 (3)	0.007 (4)

### Geometric parameters (Å, º)

**Table:**

OW—HWB	0.8500	O2—H2A	0.8200
OW—HWA	0.8502	C3—C4	1.394 (10)
Br1—C8	1.896 (7)	C3—C8	1.430 (10)
O1—C2	1.205 (9)	C4—C5	1.363 (9)
C1—O3	1.201 (9)	C4—H4A	0.9300
C1—O4	1.285 (10)	O4—H4B	0.8200
C1—C3	1.465 (10)	C5—C6	1.414 (10)
Br2—C6	1.888 (7)	C6—C7	1.363 (10)
C2—O2	1.287 (9)	C7—C8	1.367 (10)
C2—C5	1.491 (10)	C7—H7A	0.9300
			
HWB—OW—HWA	110.2	C1—O4—H4B	109.5
O3—C1—O4	122.5 (7)	C4—C5—C6	117.4 (7)
O3—C1—C3	124.2 (8)	C4—C5—C2	119.0 (7)
O4—C1—C3	113.3 (8)	C6—C5—C2	123.6 (6)
O1—C2—O2	121.4 (8)	C7—C6—C5	120.4 (7)
O1—C2—C5	123.9 (8)	C7—C6—Br2	116.2 (6)
O2—C2—C5	114.6 (6)	C5—C6—Br2	123.4 (6)
C2—O2—H2A	109.5	C6—C7—C8	121.0 (8)
C4—C3—C8	115.1 (6)	C6—C7—H7A	119.5
C4—C3—C1	120.2 (7)	C8—C7—H7A	119.5
C8—C3—C1	124.7 (7)	C7—C8—C3	121.3 (7)
C5—C4—C3	124.7 (8)	C7—C8—Br1	117.3 (6)
C5—C4—H4A	117.7	C3—C8—Br1	121.4 (5)
C3—C4—H4A	117.7		
			
O3—C1—C3—C4	−144.6 (9)	C4—C5—C6—C7	−2.9 (11)
O4—C1—C3—C4	34.9 (11)	C2—C5—C6—C7	177.8 (7)
O3—C1—C3—C8	37.0 (13)	C4—C5—C6—Br2	177.5 (5)
O4—C1—C3—C8	−143.5 (8)	C2—C5—C6—Br2	−1.9 (10)
C8—C3—C4—C5	−3.2 (11)	C5—C6—C7—C8	1.0 (12)
C1—C3—C4—C5	178.2 (7)	Br2—C6—C7—C8	−179.3 (6)
C3—C4—C5—C6	4.1 (11)	C6—C7—C8—C3	−0.1 (12)
C3—C4—C5—C2	−176.5 (7)	C6—C7—C8—Br1	179.9 (6)
O1—C2—C5—C4	169.0 (8)	C4—C3—C8—C7	1.0 (11)
O2—C2—C5—C4	−8.0 (10)	C1—C3—C8—C7	179.5 (7)
O1—C2—C5—C6	−11.7 (13)	C4—C3—C8—Br1	−178.9 (5)
O2—C2—C5—C6	171.3 (7)	C1—C3—C8—Br1	−0.4 (11)

### Hydrogen-bond geometry (Å, º)

**Table:**

*D*—H···*A*	*D*—H	H···*A*	*D*···*A*	*D*—H···*A*
O2—H2*A*···O*W*	0.82	1.74	2.554 (8)	176
O4—H4*B*···O1^i^	0.82	1.86	2.665 (8)	168
O*W*—H*WB*···O3^ii^	0.85	2.08	2.893 (9)	159
O*W*—H*WA*···O3^iii^	0.85	2.17	2.903 (9)	144
C4—H4*A*···O2	0.93	2.33	2.692 (10)	103

Symmetry codes: (i) *x*−1, −*y*+1/2, *z*−1/2; (ii) *x*+1, −*y*+1/2, *z*+1/2; (iii) −*x*+1, *y*−1/2, −*z*−1/2.
